# Interpreting success or failure of peanut oral immunotherapy

**DOI:** 10.1172/JCI155255

**Published:** 2022-01-18

**Authors:** Shijie Cao, Cathryn R. Nagler

**Affiliations:** 1 Pritzker School of Molecular Engineering and; 2 Biological Sciences Division, University of Chicago, Chicago, Illinois, USA.

## Abstract

Peanut oral immunotherapy (OIT) was recently approved by the US FDA. However, not all patients respond to OIT, and there is a high likelihood of regaining sensitization to peanuts after cessation of treatment. It is important, therefore, to identify biomarkers that impact and predict OIT outcomes. In this issue of the *JCI*, Monian, Tu, and colleagues describe distinct subsets of peanut-reactive CD4^+^ Th cell phenotypes and gene signatures with relevance to OIT outcomes using single-cell RNA-Seq and paired T cell receptor (TCR) α/β sequencing. The insights obtained will inform the development of therapeutics that target these Th cell phenotypes or deplete peanut-specific Th2 cells to achieve sustained nonresponsiveness in food allergy.

## Reducing the severity of anaphylactic reactions

Palforzia was approved by the US FDA in January 2020 as the first treatment for peanut allergy. The treatment includes daily oral administration of a controlled amount of peanut protein with escalating doses over a prolonged period to desensitize patients and reduce the severity of anaphylactic reactions (1). The treatment goal is to render patients bite-proof to accidental exposure, such that the primary efficacy endpoint is the ability to consume a challenge dose of 600 mg peanut protein without an adverse reaction (1). The treatment has a high efficacy of 67%; specifically, participants who received active treatment passed the exit food challenge (1). In addition, continued maintenance can improve the rate of efficacy (2). However, some patients do not respond to oral immunotherapy (OIT), and discontinuation can increase the likelihood of resensitization to peanuts (3). For these reasons, it is important to understand the mechanisms underlying the failure of some patients to respond to OIT.

**Figure 1:**
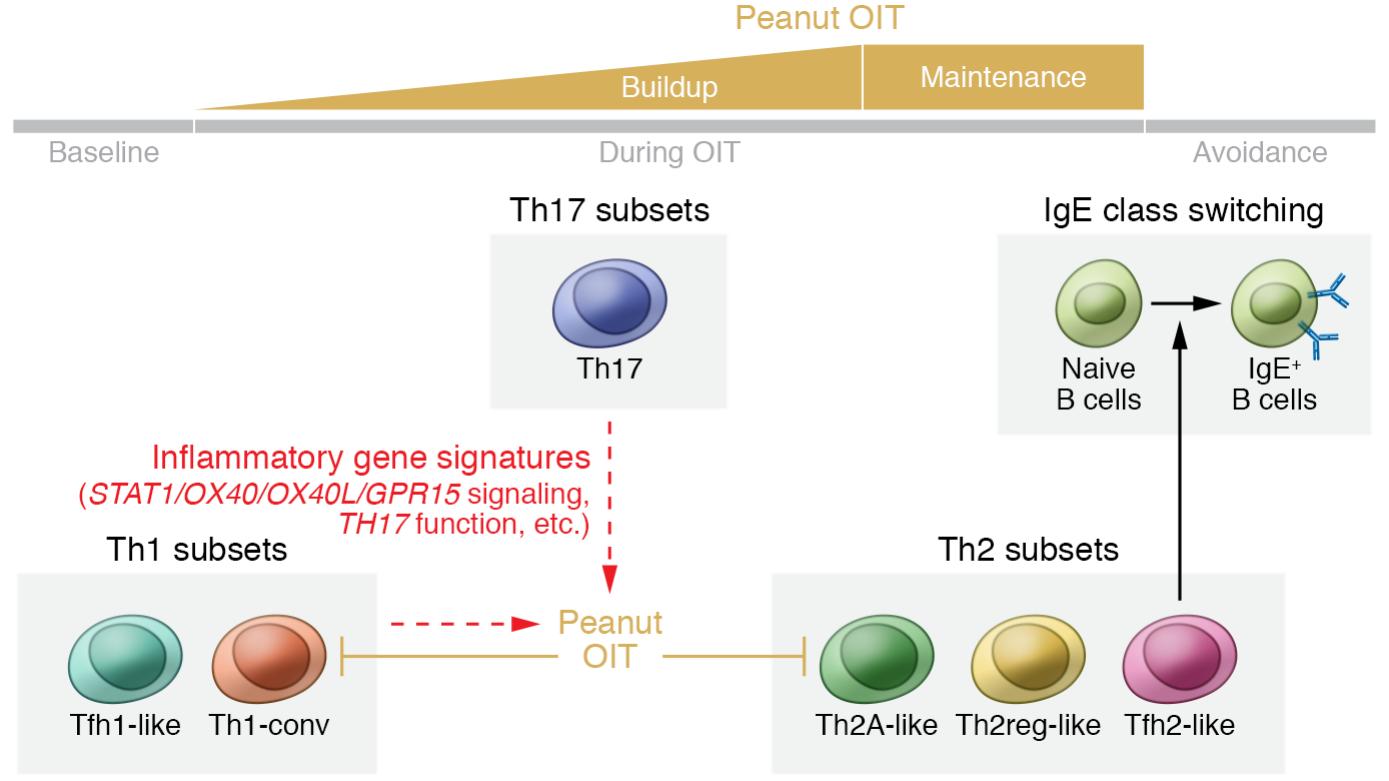
Association between clinical responses of OIT and peanut-reactive CD4^+^ T cells. Monian, Tu, and co-authors (11) assessed the transcriptomes of CD154^+^ and CD137^+^ peanut-reactive CD4^+^ Th cells from peripheral blood of patients with peanut allergy undergoing OIT. Suppression of Th1-conv and Th2A-like cell populations was associated with positive outcomes of OIT. Gene expression by Tfh2-like cells correlated with peanut-specific IgE levels, supporting the role of Tfh2 cells in class switching to IgE. Finally, the authors identified baseline inflammatory gene signatures, mostly present in Th1 and Th17 cell populations, that associated with treatment failure. These signatures suggest a potential role for these genes and Th1 and Th17 cells as predictors or influencers of OIT outcomes (dashed arrows).

Food allergies are mediated by a Th2 immune response (4, 5). The Th2 cytokines IL-4 and IL-13 promote B cell class switching to IgE; binding of allergen-specific IgE to FcεRI receptors on mast cells or basophils initiates and propagates a hypersensitivity reaction (6). OIT has been shown to suppress the circulation of Th2 effector cells (6–8). For example, Blumchen et al. reported that peanut OIT resulted in a reduction in the amount of IL-4 and IL-5 produced by PBMCs in response to stimulation with peanut extract in vitro (7). Ryan et al. demonstrated that successful OIT caused allergen-specific Th2 cells to expand and shift toward an anergic and more tolerogenic status, with increased expression of genes such as TGF-β1 (8). However, most of these findings were not correlated with the clinical outcomes of OIT treatment. Recent studies have evaluated the role of T follicular helper (Tfh) cells as an alternate source of IL-4 and IL-13 in food allergy pathogenesis (9, 10); whether OIT affects Tfh cells remains unclear. In this issue of the *JCI,* Monian, Tu, and colleagues used single-cell RNA-Seq and paired T cell receptor (TCR) α/β sequencing to analyze allergen-specific T cell populations collected from the peripheral blood of 12 patients with peanut allergy longitudinally during the course of OIT (11) (Figure 1). The authors identified distinct Th cell phenotypes and gene signatures that were relevant to OIT efficacy.

## Peanut OIT and Th cells

Monian, Tu, and co-authors first briefly activated antigen-specific T cells from the peripheral blood by in vitro stimulation with peanut extract. They then sorted peanut-reactive CD4^+^ memory T cells on the basis of CD154 and CD137 expression (11). This method enriched for antigen-specific T cells that were activated during the peanut stimulation, although the authors could not exclude the possibility that the selected cell populations still contained nonspecific, activated T cells. The T cell transcriptomes from these subsets formed distinct clusters, separated by differentially expressed genes, including *CD40LG* and *TNFRSF9*. Using sparse principal component analysis (PCA), the investigators identified gene modules that were consistent with the phenotypes of Th1, Th2, Th17, and Treg cell subsets. TCRβ was used for the subsequent clonotype analysis, since the TCRβ sequencing data covered most cells, were uniform, and paired well with a single TCRα. The TCRβ repertoire diversity of CD154^+^ and CD137^+^ cells was lower than that of the CD154^–^CD137^–^ cells. This result suggests that the CD154^+^CD137^+^ subset was associated with clonally expanded T cells activated by in vitro stimulation, effectively lessening the influence that may have derived from nonspecifically activated T cells on this analysis.

Six phenotypically distinct cell populations were further identified within Th1, Th2, and Th17 cells: Tfh2-like, Th2 regulatory–like (Th2reg-like), and Th2A-like populations within the Th2 cells, Tfh1-like and conventional Th1 (Th1-conv) cells within the Th1 cells, and one cluster of Th17 cells (ref. 11 and Figure 1). These subsets had distinct TCR repertoires, highlighting the potential role of TCR epitope interactions in skewing the T cell phenotype (12). Furthermore, the investigators identified 66 genes from Tfh2-like cells, including *IL4,* that correlated with peanut-specific plasma IgE levels, whereas gene expression in Th2A-like cells did not correlate with IgE. This correlation suggests that Tfh2-like cells may influence class switching of peanut-specific B cells to IgE. Indeed, recent findings show that IL-4–producing Tfh cells are required for IgE production and influence the affinity and longevity of antibodies produced by B cells (13–15). In addition, Gowthaman et al. have identified another subset of IL-13–producing Tfh cells that particularly associate with high-affinity IgE production (10). These IL-4– and IL-13–producing Tfh cells could complement each other to drive an anaphylactic IgE response. Notably, Tfh cells primarily reside within B cell follicles of secondary lymphoid organs, whereas the Tfh2-like cells described in the study by Monian, Tu, and co-authors (11) were identified in peripheral blood. Future studies should examine possible direct or indirect links between these Tfh2-like cells and B cell class switching.

Interestingly, the investigators assessed the impact of OIT on Th cell subtypes at different time points (i.e., at baseline and during buildup, maintenance, and avoidance; ref. 11). First, the authors showed that, while OIT decreased the abundance of peanut-reactive CD154^+^ and CD137^+^ cells in the blood overall, there was no evidence for deletion of specific TCR clones over time. However, individuals in the treatment group showed significant suppression of the Th2 module over time (*P* = 0.036). Suppression in the Th1 modules between the baseline and maintenance time points was nearly significant (*P* = 0.117), and no difference was observed for Th17 expression. The investigators then quantified gene module expression within each Th cell subset and established that Th2A-like and Th1-conv clonotypes were primarily responsible for the suppression of Th2 and Th1 gene signatures, respectively, during OIT. The suppression of Th2A-like expansion was only observed in patients who had achieved partial or full nonresponsiveness, but not in those patients who had failed the treatment or in placebo-treated patients. These data suggest that suppression of effector genes within the Th2 subset, rather than cellular depletion, could be a major factor that contributes to the success of OIT. Exhaustion or deletion of allergen-specific Th2 cells has been considered to be one of the mechanisms by which OIT alters T cell responses (16); whether or not it is key to the success of OIT remains unclear and will require further investigation.

The optimal approach to predicting OIT outcomes remains unknown and intriguing. Advances of omics technologies, such as proteomics, transcriptomics, and epigenomics, allow the identification of promising biomarkers for predicting clinical outcomes (17). Monian, Tu, and colleagues established that, although OIT had an impact on the suppression of Th2 gene signatures, the baseline composition of the Th2 subset failed to correlate with OIT outcomes (11). Thus, the authors performed PCA on the gene modules of CD154^+^ cells at baseline and attempted to stratify genes according to clinical outcome. High scores of the first principal component (PC1) were associated with poor clinical outcome, with the top five gene modules being *STAT1*, *OX40L*, *TH17*, *OX40*, and *GPR15*. Some of these genes were highly enriched in the Th1 and Th17 subsets, and, interestingly, the frequencies of Th1-conv and Th17 cells were low in the CD154^+^ group of patients for whom the treatment was successful or who achieved partial nonresponsiveness. It has previously been reported that OIT can modulate Th17 cells (18). These findings suggest a role for Th1 and Th17 cells in influencing the effectiveness of OIT (11). Unlike other reports, the authors did not find any induction of peanut-reactive Tregs during OIT after analysis of either gene expression levels or phenotypes (19, 20). Further studies with optimized methods for analyzing peanut-specific Tregs are needed to explore the predictive role of these cells in OIT.

## Conclusions and clinical implications

The study by Monian, Tu, and colleagues suggests that OIT acts predominantly via Th2A-like cell suppression rather than through clonal deletion, providing additional insight into why some patients revert to an allergic phenotype after treatment (11). Tfh2-like cell gene expression correlated with peanut-specific IgE, but OIT did not suppress Tfh2-like cells, which may provide another explanation for why it is difficult to achieve sustained nonresponsiveness through OIT. This study also established that certain gene modules broadly related to inflammation pathways at baseline were associated with the failure to respond to OIT, revealing the potential to predict success or failure of OIT before treatment begins. Future studies with larger samples and deeper sequencing approaches may reveal additional details about predictive gene signatures. Additionally, characterizing the transcriptomes of tissue-resident cell populations, particularly in the gut, will be critical to understanding how OIT influences Tfh2 cells and the resultant B cell responses. In summary, Monian, Tu, and co-authors (11) demonstrated that OIT modulated distinct Th2A-like and Th1-conv cell phenotypes and identified gene signatures that could potentially predict OIT efficacy. These clues to cellular mechanisms of OIT may provide insight into targets for the treatment of food allergy.

## Version 1 01/18/2022

Electronic publication
